# Correction: Determining engineering properties of ultra-high-performance fiber-reinforced geopolymer concrete modified with different waste materials

**DOI:** 10.1371/journal.pone.0333548

**Published:** 2025-09-30

**Authors:** Fadi Althoey, Osama Zaid, Saleh Alsulamy, Rebeca Martínez-García, Jesús de Prado Gil, Mohamed M. Arbili

In the Funding section, the grant number from the King Khalid University is listed incorrectly. The correct grant number is: RGP.2/262/44.
